# The Practical Possibilities of Cancer Research

**Published:** 1912-06-22

**Authors:** 


### The Hospital

The Workers' Newspaper of Administrative Medicine and Institutional Life, Administration, National Insurance and Health.

No. 1355, Vol. LII. SATURDAY, JUNE 22, 1912. PRICE ONE PENNY.

### THE PRACTICAL POSSIBILITIES OF CANCER RESEARCH.

The announcement recently made that Professor Wassermann, who is well known in connection with his investigations in syphilology, is engaged upon the task of finding a chemical compound that shall have the same antagonistic selective action upon the cancer cell as salvarsan is said to have upon the spirochæte of syphilis will be cordially welcomed by those who believe that cancer research has hitherto been deviated too much from clinical and practical into theoretical paths. During the last ten years a large amount of time, money, and energy has been expended in laboratory work which is dignified by the name of "cancer research" with comparatively little practical result. Indeed, it is hardly exaggeration to say that this expenditure has produced no new fact of any outstanding importance, and has not increased our knowledge of the disease in a degree commensurate with the expectations and the hopes held out when the combined attack upon the problem was initiated. Every year elaborate reports are published which to the unprejudiced reader, who is moreover acquainted with the history of the study of cancer, seem curiously redundant, and marked more by insistent tautological assertion on matters of subsidiary importance than by the evidence of original work of real value. We say this deliberately, and with no intention of belittling the enthusiastic and painstaking efforts of those who are responsible for the reports. But the matter is one in discussing which plain-spoken terms are best, and there can be little doubt that the extravagant eulogy annually devoted to the work that has been accomplished during the preceding twelve months is scarcely warranted by the importance of that work. At present the efforts of the investigators are devoted to investigation of the kind that is preferably left to individual enthusiasm, and it is unlikely that by pursuing such laboratory experiments, the main object of which is to demonstrate some pathological theory which is founded on the vaguest surmises, the elucidation of the ætiology of cancer will be materially furthered. That being the case, it is worth while to question whether it is altogether justifiable that the public should be appealed to so constantly to support this line of investigation to the neglect of other and presumably more practical methods of the kind initiated by Professor Wassermann, Dr. Coley, and those who are occupying themselves with what may be called the serum treatment of the disease.

It is this latter line of investigation that holds out more promise of success: it is this, too, that by its very nature should appeal to the public. We have always insisted that the problem of cancer must be attacked from a clinical and not merely from a laboratory point of view, and that the results obtained in the ward in the way of facts, statistics, and experience are far more important and valuable than those which are to be derived from experiments on animals. The experimental method, as conducted in the research laboratories, has produced little that is new; almost every conclusion drawn from it has been previously deduced from bedside observation and clinical experience. Moreover, some of the findings are in direct opposition to the conclusions legitimately to be drawn from a careful study of cancer in the human subject; for example, the possibility of a hereditary predisposition to the disease. The parasitic theory of cancer is not one that is favoured by the laboratory investigators, yet only recently the late Sir Henry Butlin showed how strong a case for that hypothesis could be made out.

A great deal of excellent work on the subject of the disease is being done at every cancer hospital and in every ward that contains cancer patients in a general hospital. The data regarding this work are buried in hospital and clinical reports: only the energy of some enthusiastic volunteer can draw attention to important points of incidence, pathology, diagnosis, and treatment which are apt to be overlooked. By systematising this investigation into hospital records it may be possible to gain much new information which would otherwise not be available for discussion. It seems to us that an attempt to work on these lines may be productive of far more good than the isolated work in the labora tory on the cytology of the disease and the question of its communicability to mice. Necessarily the two lines of research should be proceeded with a the same time—the problem cannot be attacked from too many sides—and co-operation between the investigators is imperative if the data collected are to be made available for examination. Hitherto, however, the tendency has been to neglect the therapeutic and clinical aspect of the problem. We are fully aware that there are brilliant exceptions. The therapeutic work carried on in the wards of the Middlesex Hospital, for instance, has negatively increased our knowledge to a considerable extent, but even this work has been carried on in an unsystematic and irregular fashion. The investigation of those rare cases of spontaneous cure, or apparent cure, of cancer is above all one of the subjects that imperatively demands attention. So far as we know, no combined attack has been made on these cases to find out the causes for the disappearance of the tumour. Yet, arguing by analogy, it is extremely likely that the practical possibilities of cancer research, so far as the discovery of a cure for the disease is concerned, lie in this direction. At least the subject is one which is worth all the attention that can be given to it. And a little of the superfluous energy devoted to observing the growth of cancer cells in the lower animals and in the laboratory generally may with advantage be devoted to supplying this attention to the study of one of the most fascinating aspects of the cancer problem. The interest excited in a certain libel case makes a reminder of this kind not inopportune.

